# Study on the Inhibition Mechanism of Hydration Expansion of Yunnan Gas Shale using Modified Asphalt

**DOI:** 10.3390/ma17030645

**Published:** 2024-01-29

**Authors:** Zhiwen Dai, Jinsheng Sun, Jingping Liu, Kaihe Lv, Xianfa Zhang, Zonglun Wang, Zhe Xu

**Affiliations:** 1 Key Laboratory of Unconventional Oil & Gas Development, China University of Petroleum (East China), Ministry of Education, Qingdao 266580, China; 2 School of Petroleum Engineering, China University of Petroleum (East China), Qingdao 266580, China; 3 CNPC Engineering Technology R&D Company Limited, Beijing 102206, China

**Keywords:** inhibition, shale gas, modified asphalt, water-based drilling fluids

## Abstract

Drilling fluids play an essential role in shale gas development. It is not possible to scale up the use of water-based drilling fluid in shale gas drilling in Yunnan, China, because conventional inhibitors cannot effectively inhibit the hydration of the illite-rich shale formed. In this study, the inhibition performance of modified asphalt was evaluated using the plugging test, expansion test, shale recovery experiment, and rock compressive strength test. The experimental results show that in a 3% modified asphalt solution, the expansion of shale is reduced by 56.3%, the recovery is as high as 97.8%, water absorption is reduced by 24.3%, and the compression resistance is doubled compared with those in water. Moreover, the modified asphalt can effectively reduce the fluid loss of the drilling fluid. Modified asphalt can form a hydrophobic membrane through a large amount of adsorption on the shale surface, consequently inhibiting shale hydration. Simultaneously, modified asphalt can reduce the entrance of water into the shale through blocking pores, micro-cracks, and cracks and further inhibit the hydration expansion of shale. This demonstrates that modified asphalt will be an ideal choice for drilling shale gas formations in Yunnan through water-based drilling fluids.

## 1. Introduction

The demand for oil and gas is significantly increasing, and the annual output of conventional oil and gas reservoirs is declining. Therefore, oil engineers are turning their attention to oil and gas in unconventional resources [1,2,3]. The unconventional energy source represented by shale gas is quietly changing the world energy landscape and has become the focus of global oil companies [4,5]. Shale gas has the advantages of a large resource potential, wide distribution range, high cleanliness, and high efficiency [6,7]. In 2022, China’s external dependence on crude oil was 71.2% and its dependence on natural gas was 40.5%. Therefore, the efficient development of shale gas is conducive to safeguarding China’s energy security.

Drilling fluids play a crucial role in the development of shale gas [8,9]. The main functions of the drilling fluid are to clean the well bottom, balance the formation pressure, stabilize the well wall, suspend weighting materials and cuttings, cool and lubricate the drill bit, and transmit power [10]. Shale gas reservoirs in China are abundant [11]. Oil-based drilling fluids have made outstanding contributions to shale gas drilling in Yunnan, China [12]. On the one hand, they have the advantages of strong inhibition and high lubricity, and on the other hand, they have the disadvantages of high cost and environmental pollution. Water-based drilling fluids have the advantages of low cost and environmental friendliness and are currently the main research direction [13].

There are nano micro-pores and micro-cracks in the shale gas formation of Yunnan, and the formation is rich in organic matter and clay minerals and has strong heterogeneity [14]. The filtrate of the water-based drilling fluid passes through the pores, micro-cracks, or cracks in the shale formation during drilling, and the clay mineral in the formation hydrates and swells, resulting in several accidents such as wellbore collapse and a stuck pipe during the drilling operation. These accidents hinder the large-scale application of water-based drilling fluids in shale gas drilling [15,16].

The addition of an inhibitor to the drilling fluid is a commonly used effective method to inhibit the expansion of clay minerals due to hydration [17]. Silicates can inhibit the shale hydration expansion through blocking the pores of the formation and adsorbing on the surface of the formation rock to form an adsorption membrane [18,19]. Potassium chloride (KCl) and sodium chloride (NaCl) inhibit the hydration of clay through compressing the diffusion double layer of clay minerals produced in water [20,21]. The sources of NaCl are widespread, its cost is low, and the potassium ion also has a blocking performance. Potassium formate solution has the characteristics of high salinity, high liquid viscosity, low water activity, and low surface tension, which enables potassium formate to effectively reduce the hydration of clay minerals and stabilize wellbore stability [22]. With a unique molecular structure, polyamines can penetrate into the layers of clay minerals and bind them together to effectively reduce clay hydration [23]. Aluminium-based polymers can block the pores of the formation by precipitation with the calcium and magnesium ions in the formation fluid, preventing the drilling fluid from invading the formation.

A variety of nanomaterials can inhibit shale hydration by adsorbing on the surface of the formation, and they can also block the nanopores of the shale, thus preventing the filtrate of the drilling fluid from entering the formation [24,25]. Asphalt has the potential to be used as an additive in drilling fluids because it can plug pores, form dense mud cakes, and reduce filtration loss [26]. To further improve the dispersibility of asphalt, asphalt shale inhibitors such as sulfonated asphalt and emulsified asphalt have been developed [27]. Xionghu et al. prepared nano asphalt particles with cationic groups, improving the adsorption and blocking properties of asphalt powder [28]. However, owing to the poor matching between the existing conventional inhibitors and Yunnan shale formations, wellbore instability caused by the hydration expansion of the formation is still severe. Therefore, water-based drilling fluids are facing huge challenges in Yunnan shale formation. It is crucial to develop high-efficiency inhibitors that can effectively inhibit the swelling of shale formations in Yunnan.

## 2. Materials and Methods

### 2.1. Materials

Sodium chloride (96%), potassium chloride (99.5%), sodium silicate (98%), and potassium silicate (99%) were provided by Sinopharm Chemical Corporation (Shanghai, China). The shale samples used for the tests were collected in Yunnan (China). The modified asphalt was prepared by sulfonation of asphalt powder and styrene modifier with fuming sulfuric acid.

### 2.2. Equipment

The experimental equipment used in the tests is shown in Table 1.

### 2.3. Mineralogical Composition Analysis

An analysis of the constitutive characteristics of the shale is a prerequisite for studying the shale hydration expansion characteristics and hydration inhibitors. The shale from the Longmaxi Formation in Yunnan was selected for study and the shale composition was analysed using X-ray diffraction (SY/T 5163-2010).

### 2.4. Inhibition Performance Test

#### 2.4.1. Plugging Test

The permeability of the shale samples was measured using the pulse decay method. First, after setting the confining pressure to 13.79 MPa and the saturation pressure to 6.89 MPa, a core with a diameter of 25 mm and a length of 50 mm was saturated with nitrogen for 24 h. The initial permeability (K_0_) of the shale sample was then determined through a pulse attenuation permeameter. Second, at the displacement pressure of 3.5 MPa and confining pressure of 5 MPa, a single end of the shale sample was sealed with a modified asphalt solution at room temperature using a core flow meter for 3 h. Finally, the shale sample was dried at room temperature until constant weight was attained, and the permeability (K_f_) of the sample was measured again using the pulse method:

(1) $$\mathrm{Blocking}=\frac{K_{o}-K_{f}}{K_{0}}\text{×}100\%$$

#### 2.4.2. Expansion Test of Shale

The shale expansion test can evaluate the inhibitory properties of various inhibitors on shale hydration expansion, which is advantageous for inhibitors that are suitable for different shales. In this study, the shale was pulverized into a powder with a particle size of less than 100 mesh using a pulverizer. A series of expansion experiments were then conducted on the shale in inhibitor solutions of different concentrations and temperatures through a high-temperature and high-pressure dilatometer.

#### 2.4.3. Shale Recovery Test

Shale recovery experiments can evaluate the inhibitory properties of various inhibitors on shale hydration dispersion, favouring inhibitors that are suitable for different shales [29]. First, the shale sample is pulverized into particles with a particle size of 6–10 mesh, and 30 g of shale particles are placed in an aging tank containing 300 mL of inhibitor solution in a high-temperature roller heating furnace for hot rolling at 100 °C for 16 h. Then, particles having a diameter of more than 40 mesh sieves were selected and washed with water. Finally, after drying at 80 °C, the weight of the particles was recorded as M_1_:

(2) $$\mathrm{Recovery}=\frac{M_{1}}{30}\text{×}100\%$$

#### 2.4.4. Rock Compressive Strength Test

During the drilling process, after the shale formation meets the water, the clay minerals in the shale are hydrated to expand or disperse, which reduces the strength of the shale in the wellbore. The more the shale strength decreases, the higher the risk of collapse of the wellbore during drilling. Therefore, it is necessary to add an inhibitor to the drilling fluid to maintain the compressive strength of the shale. In this study, shale cores with a length of 30 mm and a diameter of 25 mm were selected, and the compressive strength was measured after soaking them for 24 h in different inhibitor solutions at different temperatures using a TAW-2000 rock triaxial tester with an axial deformation rate of 0.00125 mm/s.

### 2.5. Mechanism Analysis

#### 2.5.1. Water Absorption Test

The water absorption of the shale can affect its stability. The more water the shale absorbs, the greater the chance of accidents in the shale wall during drilling, and the more unsafe the drilling. The water absorption test can help screen out inhibitors that help reduce the water absorption of shale and stabilize the well wall. A cylindrical core sample, 25 mm in diameter and 25 mm in height, was prepared. The sample was drilled perpendicular to the bedding. The shale sample was dried at 70 °C until constant weight was obtained, and then cooled down in a desiccator under vacuum at room temperature, which was finally de-watered. After that, the sample was immersed in water by placing it in a glass jar. The water level in the jar remained constant at approximately 15 mm above the top of the sample during this experiment. At the end of a certain duration, the mass of the sample was measured using a balance, and then the amount of absorbed water was calculated and normalized with respect to the weight of the specimen. The amount of water absorption was calculated (Equation (3)):

(3) $$A=(W_{\mathrm{sat}\;}-\;W_{\mathrm{dry}})/W_{\mathrm{dry}},$$

where A is the water absorption per gram of shale (g/g), W_sat_ is the weight of the sample saturated in water (g), and W_dry_ is the weight of the oven-dried sample (g).

#### 2.5.2. Scanning Electron Microscope (SEM) Analysis

The pores, micro-cracks, and cracks in the shale are the main channels for the water in the drilling fluid to enter the deep part of the formation. Their shape, size, and distribution determine the difficulty of the drilling process. In this study, the morphology of pores, micro-cracks, and cracks in the shale was observed using a JEOL JSE-6510 SEM (Tokyo Metropolis, Japan). The scanning voltage was 3 kV.

The microscopic morphology of the shale before and after immersion in the modified asphalt solution can reflect the interaction between the shale and the modified asphalt. In this study, the shale was immersed in a 3% modified asphalt solution for 24 h, and then naturally dried in a drying dish for 24 h. Then, the morphology of the shale was observed with SEM after the gold was sprayed on the shale [30].

#### 2.5.3. Modified Asphalt Adsorption Experiment

The adsorption amount can effectively reflect the interaction between the shale and the modified asphalt. The larger the adsorption of the modified asphalt on the shale was, the stronger its inhibition effect on the shale. The shale was placed in a modified pitch solution at 25 °C, and the adsorption amount of the modified asphalt on the shale was measured using a molecular fluorescence spectrophotometer [31]. The adsorption time was 24 h. When the adsorption amount was measured using the instrument, the concentration of the modified asphalt in the aqueous solution was too high, which made the colour of the solution too dark, thus affecting the fluorescence inspection and resulting in inaccurate measurement results. Hence, the experiment can only be carried out with a low concentration of modified asphalt solution.

#### 2.5.4. Contact Angle Measurement

The change in contact angle of water droplets on the shale before and after immersion in the modified asphalt solution can reflect the influence of modified asphalt on the wettability of the shale surface and, thus, affect the hydration performance of the shale. In this study, the shale was immersed in a 3% modified asphalt solution for 24 h, then naturally dried in a drying dish for 24 h, and the contact angle of the water droplets of the shale was observed using an optical contact angle meter.

### 2.6. Performance of Drilling Fluid

Modified asphalt can be applied to shale gas drilling depending on whether it matches with other treatment agents in the drilling fluid and does not affect the performance of these other agents. In this study, modified asphalt was added into a set of drilling fluid formulations, and their basic properties, such as rheology and fluid loss, were measured before and after aging at 100 °C using the API recommended procedure for the testing of drilling fluids [32,33].

## 3. Results and Discussion

### 3.1. Mineralogical Composition Analysis

**Figure 1:**
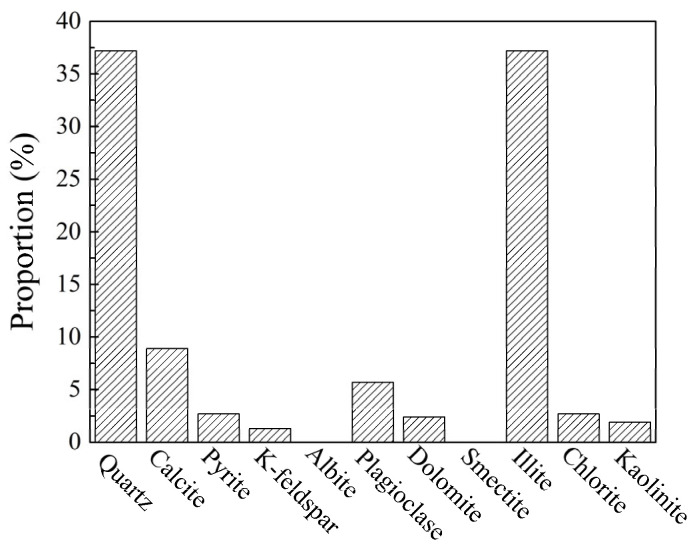
Mineralogical composition analysis of Yunnan shale.

As shown in Figure 1, the non-clay minerals in Yunnan shale were mainly quartz and calcite, with contents of 37.2% and 8.9%, respectively. The content and type of clay minerals determined the hydration performance of shale. The content of clay mineral in Yunnan shale was relatively high, reaching 41.8%, and thus, the hydration performance of this shale was strong. The clay mineral was illite, with a content of 37.2%, and did not contain montmorillonite.

### 3.2. Inhibition of Shale Hydration by Modified Asphalt

#### 3.2.1. Plugging Test

As presented in Table 2, after treatment of the shale cores with water, the shale permeability increased from 20.46 × 10^−7^ μm^2^ to 38.52 × 10^−7^ μm^2^, which may be due to the dispersion of the hydrated clay minerals or dissolution of the salt component in the shale. After plugging it with a modified asphalt solution, the permeability of the shale was significantly reduced. When the concentration of modified asphalt was increased from 0.5% to 4%, the blocking increased from 62.7% to 96.89%, indicating that modified asphalt can effectively block shale voids, micro-cracks, and cracks, inhibit water from entering the shale, and stabilize the borehole wall.

#### 3.2.2. Expansion Test of Shale

**Figure 2:**
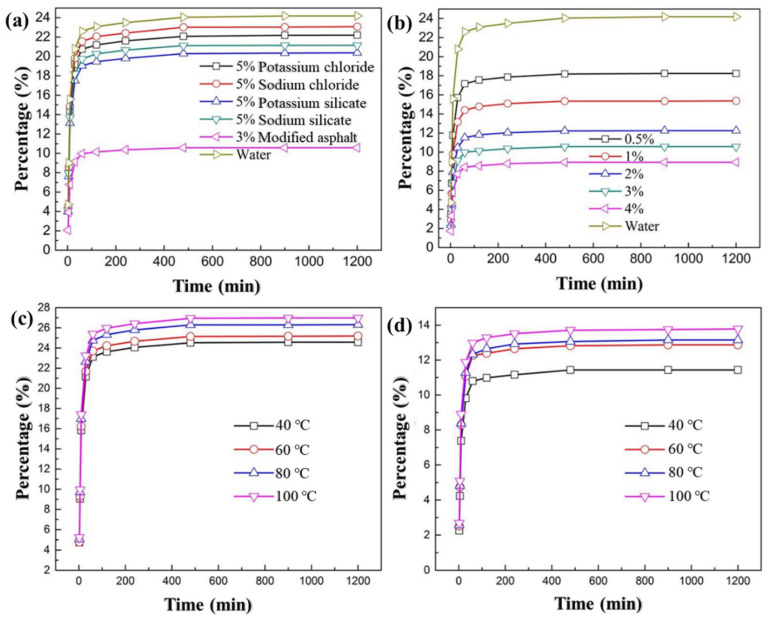
(**a**) Expansion of shale in different inhibitor solutions. (**b**) Expansion of shale in solutions of modified asphalt with different concentrations. (**c**) Expansion of shale in water at different temperatures. (**d**) Expansion of shale in a 3% modified asphalt solution at different temperatures.

As shown in Figure 2a, the expansions of shale after 1200 min in water, 5% potassium chloride, 5% sodium chloride, 5% potassium silicate, and 5% sodium silicate solution were 24.19%, 22.21%, 23.07%, 20.38%, and 21.15%, respectively. In the potassium chloride, sodium chloride, potassium silicate, and sodium silicate solutions, the shale expansion rate decreased slightly, indicating that these commonly used inhibitors had poor inhibition of shale gas formations in Yunnan. However, in the 3% modified asphalt solution, the expansion of shale was only 10.57%, which was 13.62% lower than that in water, indicating that the modified asphalt had a good inhibitory on the hydration expansion of shale. The expansions of the shale at 60 min in water, 3% modified asphalt, 5% potassium chloride, 5% sodium chloride, 5% potassium silicate, and 5% sodium silicate solutions were 22.61%, 9.97%, 20.75%, 21.56%, 19.04%, and 19.75%, respectively. They were close to the final shale expansions at 1200 min, indicating that the main hydration expansion process of the shale occurred before the first 60 min and the shale hydration was a rapid process, which may be related to the fact that the main clay mineral in the shale was illite. Illite underwent surface hydration, which was a rapid process.

As shown in Figure 2b, when the concentration of modified asphalt in the solution increased from 0.5% to 4%, the shale expansion changed from 18.24% to 8.93%, indicating that the modified asphalt had a strong inhibitory effect on the hydration expansion of Yunnan shale, and the inhibition was stronger as the concentration increased. From the perspective of cost and dosage, 3% was selected as the optimum addition amount of modified asphalt.

During the drilling process, the hydration expansion of the shale was carried out under specific formation temperature conditions. Therefore, it was more accurate to evaluate the inhibition performance of modified asphalt on shale expansion under different temperature conditions. It was determined whether modified asphalt can be used in shale gas drilling. When the temperature was raised from 40 °C to 100 °C (Figure 2c), the shale expansion increased from 24.57% to 26.98%, indicating that the elevated temperature exacerbated the hydration expansion of the shale. In the 3% modified asphalt solution, the shale swelling changed from 11.43% to 13.78% (Figure 2d), indicating that the modified asphalt can effectively inhibit the hydration expansion of shale at different temperatures, which proves that modified asphalt can be used for drilling in shale gas formations under high temperatures.

#### 3.2.3. Shale Recovery Test

**Figure 3:**
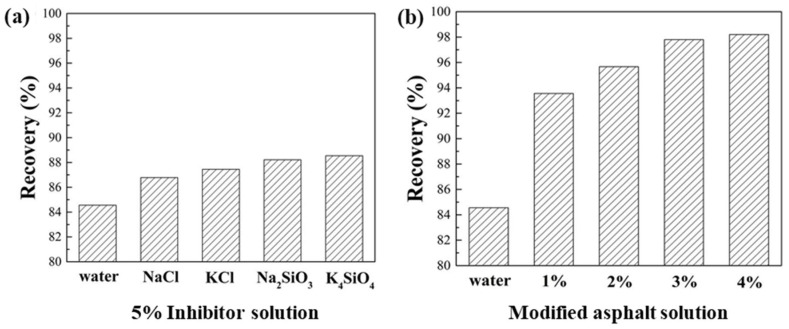
(**a**) Shale recovery tests for shale with different 5% inhibitor solutions after 100 °C hot rolling. (**b**) Shale recovery tests for shale in solutions with different concentrations of modified asphalt after 100 °C hot rolling.

The recovery test can evaluate the probability of a fall of the shale wall during drilling in a shale gas formation. As shown in Figure 3a, in water, 5% sodium chloride, 5% potassium chloride, 5% sodium silicate, and 5% potassium silicate solutions, the recovery rates of the shale were 84.56%, 86.78%, 87.45%, 88.21%, and 88.54%, respectively. The increase in shale recovery was small, indicating that these commonly used inhibitors had a poor inhibitory effect on shale gas formation dispersion. As shown in Figure 3b, when the concentration of modified asphalt increased from 1% to 4%, the shale recovery increased from 93.56% to 98.20%, indicating that modified asphalt had a good inhibitory effect on shale dispersion, and with increasing concentration, the inhibition became stronger.

#### 3.2.4. Rock Compressive Strength Test

**Figure 4:**
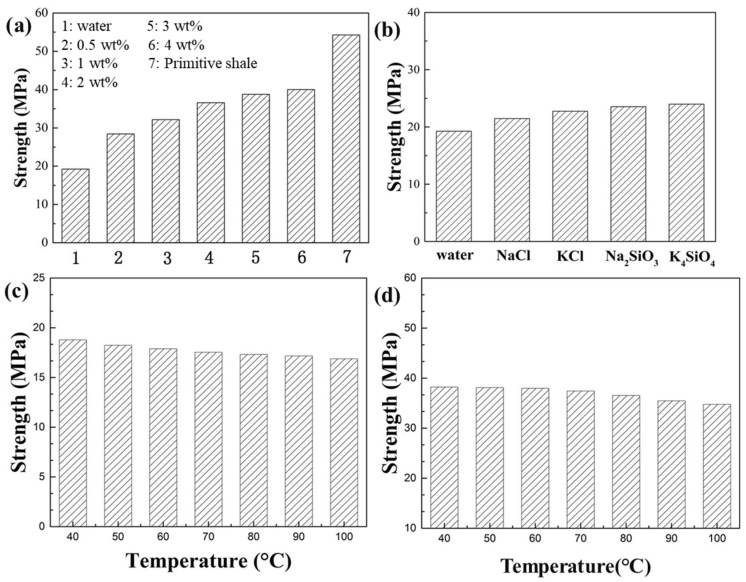
(**a**) Shale strength after immersion in different concentrations of modified asphalt solutions. (**b**) Shale strength after immersion in commonly used inhibitor solutions with 5% concentration. (**c**) Shale strength after immersion in water at different temperatures. (**d**) Shale strength after immersion in a 3% modified asphalt solution at different temperatures.

As shown in Figure 4a, after the shale was immersed in water, the compressive strength of the shale was reduced from 54.26 to 19.25 MPa. This was due to the hydration of clay in the shale, which considerably reduced its compressive strength. When the concentration of modified asphalt in water increased from 0.5% to 4%, the compressive strength of the shale increased from 28.44 to 40.03 MPa, indicating that the modified asphalt effectively maintained the compressive strength of the shale. This was because a large amount of modified asphalt can be adsorbed on the surface, making the shale surface more hydrophobic.

As shown in Figure 4b, after the shale was immersed in sodium chloride, potassium chloride, sodium silicate, and potassium silicate solutions at a concentration of 5% for 24 h, the compressive strengths were 21.46, 22.75, 23.54, and 23.98 MPa, respectively. The increase in compressive strength was not effective, indicating that conventional inhibitors cannot effectively maintain the compressive strength of the shale because they cannot effectively inhibit the Yunnan shale hydration.

As shown in Figure 4c, after immersion in water at a temperature range of 40–100 °C, the shale compressive strength was reduced from 18.78 to 16.88 MPa, indicating that the elevated temperature reduced the compressive strength. Thus, a high temperature was more detrimental to the stability of the borehole wall, which may be related to the promotion of hydration of clay minerals in shale at high temperatures.

As shown in Figure 4d, after immersion in a 3% modified asphalt solution at a temperature range of 40–100 °C, the shale compressive strength was reduced from 38.23 to 34.78 MPa. Although the compressive strength of the shale in the modified asphalt solution decreased with increasing temperature, the reduction was not large, and the modified asphalt still effectively maintained the compressive strength of the shale, which was conducive to the stability of the well wall.

### 3.3. Mechanism Analysis

#### 3.3.1. Water Absorption Test

**Figure 5:**
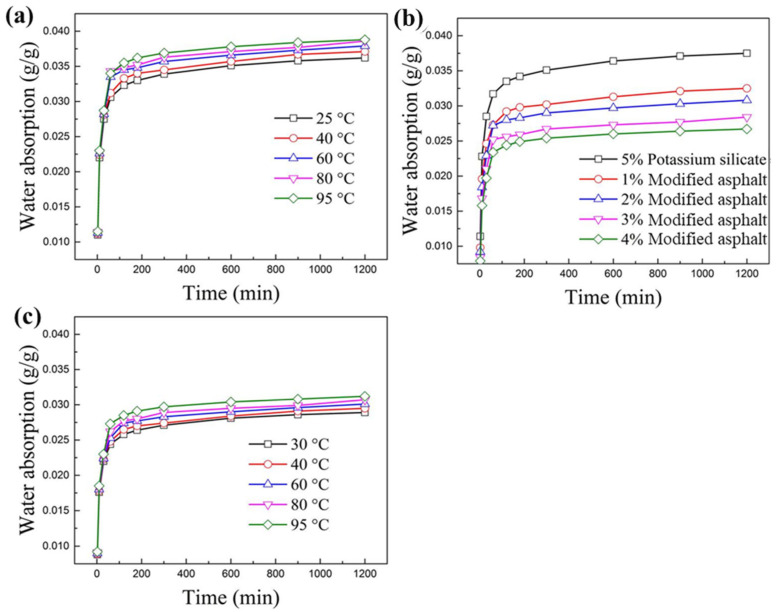
(**a**) Water absorption of the shale at different temperatures. (**b**) Water absorption of the shale in sulfonated asphalt solutions with different concentrations at 25 °C. (**c**) Water absorption of the shale in a 3% sulfonated asphalt solution at different temperatures.

When the temperature was raised from 25 °C to 95 °C, the water absorption of the shale in water increased from 0.0362 to 0.0388 g/g. The higher the temperature, the greater the water absorption of the shale, indicating that an elevated temperature promoted water absorption of the shale (Figure 5a), which was assumed to be related to the promotion effect of a high temperature on the hydration expansion of the shale. In a 5% potassium silicate solution, the water absorption was 0.0375 g/g, which is higher than the absorption in water at 25 °C. Potassium silicate did not inhibit water absorption of the shale, but promoted it, possibly because potassium silicate was heavily adsorbed on the surface of the shale, increasing its weight. However, in the modified asphalt solution, the water absorption of the shale was significantly reduced. When the concentration of modified asphalt increased from 1% to 4%, the water absorption of the shale decreased from 0.0325 to 0.0267 g/g, indicating that the modified asphalt inhibited water absorption, and the higher the concentration was, the stronger the inhibition (Figure 5b). In a 3% modified asphalt solution, the water absorption of the shale in water varied from 0.0289 to 0.0312 g/g when the temperature was raised from 30 °C to 95 °C, indicating that modified asphalt can effectively control the water absorption of the shale at different temperatures (Figure 5c).

#### 3.3.2. Scanning Electron Microscope (SEM) Analysis

**Figure 6:**
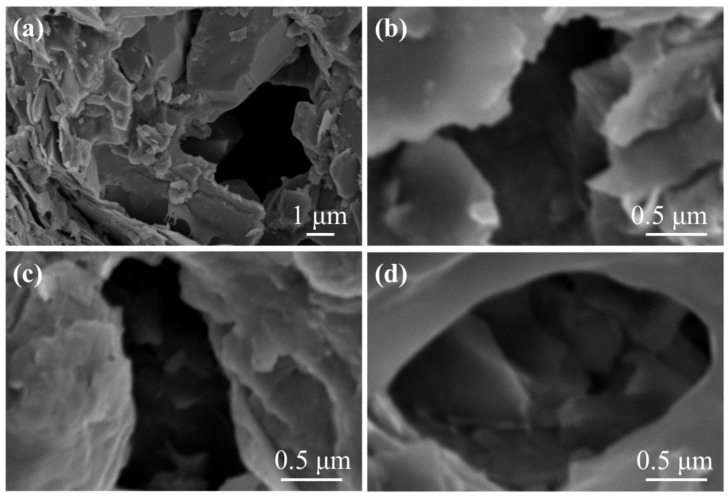
SEM images of pores in the shale: (**a**) The width of pores rang from 1 to 4 μm. (**b**) Presence of quartz in the hole. (**c**) Laminate clay minerals near the pore edges. (**d**) Dissolution pore.

Figure 6 shows four different pores in the shale in Yunnan. The width of these pores ranged from 1 to 4.5 μm, and their shapes were different, with the laminate structure of clay minerals near the pore edges. When drilling with water-based drilling fluids, these pores provided access for water to enter the shale.

**Figure 7:**
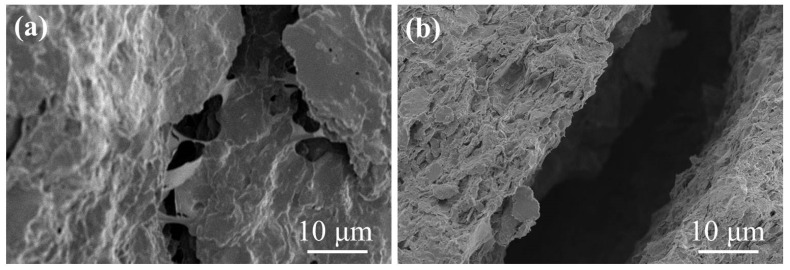
SEM images of micro-cracks in the shale: (**a**) Intersecting micro-cracks. (**b**) Micro-crack along the interface of the layer system.

The shape of a set of intersecting micro-cracks in the shale is shown in Figure 7a, and their width varied from 5.5 to 10 μm. Figure 7b shows a micro-crack along the interface of the layer system with a width from 25 to 30 μm. These micro-cracks provided access for water to enter the shale when drilling. Owing to the existence of these pores, micro-cracks, and cracks, it was difficult to stabilize the borehole wall during the drilling.

**Figure 8:**
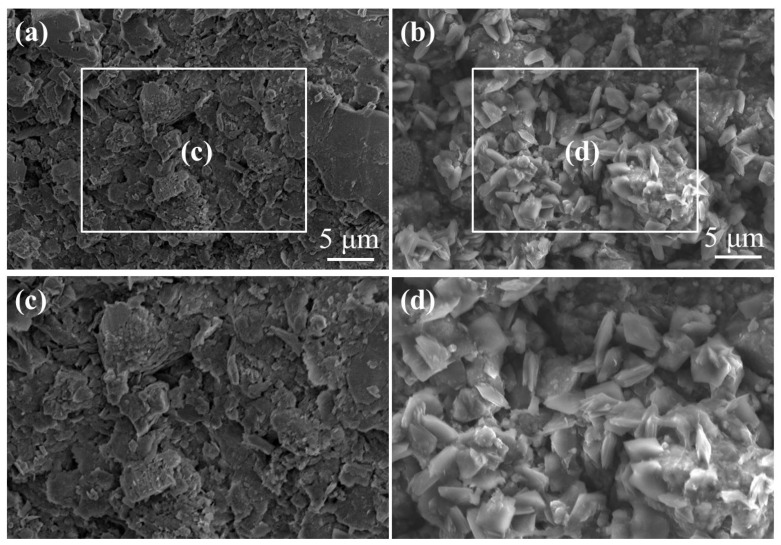
(**a**) SEM image of the original shale. (**b**) SEM image of the shale after immersing in 3% modified asphalt solution. (**c**) Magnification of the boxed area in (**a**). (**d**) Magnification of the boxed area in (**b**).

Figure 8a shows that the original shale had many pores in the surface and was accompanied by several layered stacked structures. After immersing it in a 3% modified asphalt solution, as shown in Figure 8b, the flaky modified bitumen particles underwent a large amount of adsorption on the surface of the shale, and a protective layer was formed. This could block the pores, micro-cracks, and cracks in the shale with the effect of changing the wettability of the shale surface, which is beneficial to stabilize the shale formation in the wellbore.

#### 3.3.3. Modified Asphalt Adsorption Experiment

**Figure 9:**
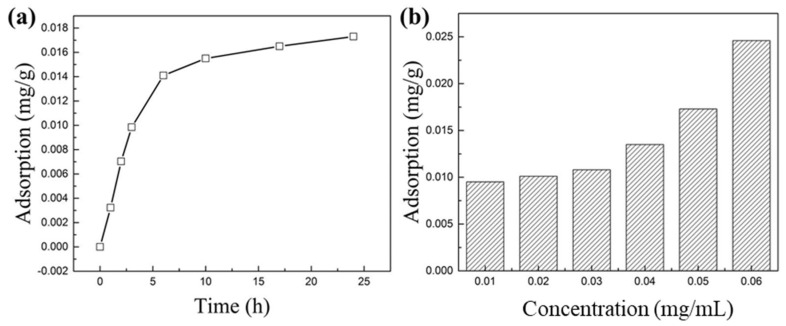
(**a**) Adsorption amount as a function of time (25 °C). (**b**) The adsorption amount varies with the concentration of modified asphalt (25 °C).

The adsorption amount of modified asphalt on the shale increased rapidly within 0 to 6 h and increased slowly after 6 h. It reached 0.0173 mg/g after 24 h in a modified asphalt solution with a concentration of 0.05 mg/mL, as shown in Figure 9. When the concentration of modified asphalt changed from 0.01 to 0.06 mg/mL, the amount of modified asphalt adsorbed on the shale increased from 0.0095 to 0.0246 mg/g, indicating that the increase in concentration would promote the adsorption of modified asphalt. Therefore, the increase in concentration will enhance the shale inhibition by the modified asphalt.

#### 3.3.4. Contact Angle Measurement

**Figure 10:**
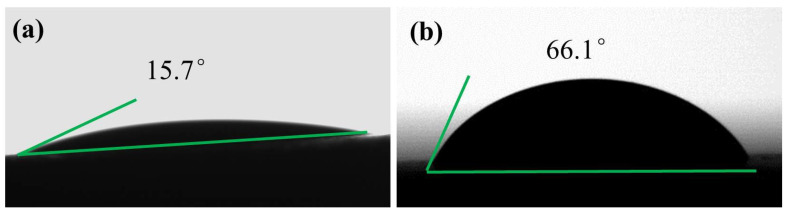
(**a**) Water droplet contact angle of shale. (**b**) Water droplet contact angle of shale after immersion in 3% modified asphalt solution.

Figure 10 shows that the contact angles of the shale before and after immersion in a 3 wt% modified asphalt solution were 15.7° and 66.1°, respectively, indicating that the modified asphalt enhanced the hydrophobicity of the shale. It reduced the surface free energy of the shale, thus weakening the ability of the shale surface to adsorb water molecules, inhibiting hydration of the shale surface, and stabilizing the shale.

**Figure 11:**
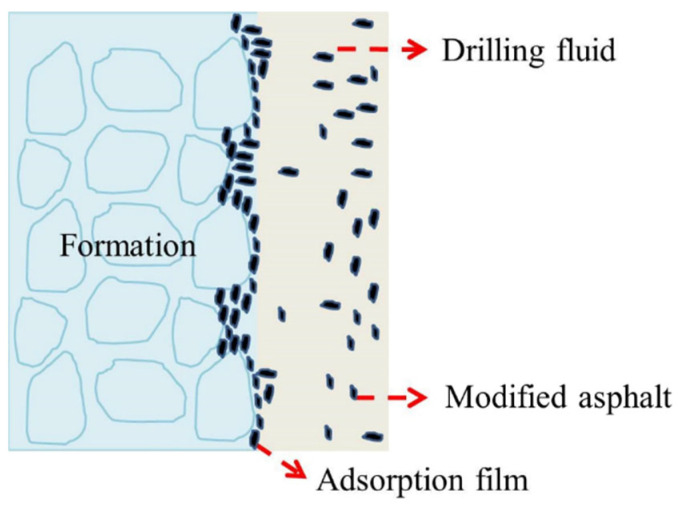
Mechanism of modified asphalt inhibiting shale hydration.

As shown in Figure 11, the modified asphalt was adsorbed on the surface of the shale to form a hydrophobic membrane, which is the driving force of water adsorption on the shale surface. Simultaneously, the modified asphalt can block the shale pores, micro-cracks, and cracks in the shale formation, preventing the filtrate in the drilling fluid from entering the shale, thereby further inhibiting shale hydration and stabilizing the well wall.

### 3.4. Application of Modified Asphalt-Containing Drilling Fluid

The drilling fluids composition is shown in Table 3. The drilling fluids were weighted with barite to a density of 2.0 g/cm^3^.

As presented in Table 4, after adding modified asphalt, the plastic viscosity (PV), yield point (YP), and gel strength of the drilling fluid did not change much, indicating that the modified asphalt did not damage the rheology of the drilling fluid. After adding 3% modified asphalt, the API fluid loss as well as the high-temperature and high-pressure fluid loss of the drilling fluid were significantly reduced, from 0.6 mL and 5.4 mL to 0.2 mL and 4.0 mL, respectively, indicating that the addition of modified asphalt reduces the fluid loss of the drilling fluid. This was because the modified asphalt can effectively block the mud cake formed by the fluid loss of the drilling fluid, making the mud cake dense, and thus, the fluid loss was considerably reduced. Modified asphalt can effectively cooperate with the treatment agent in the drilling fluid, so that the performance of the drilling fluid was better, and that it can be fully applied to the drilling of the shale gas formation.

**Figure 12:**
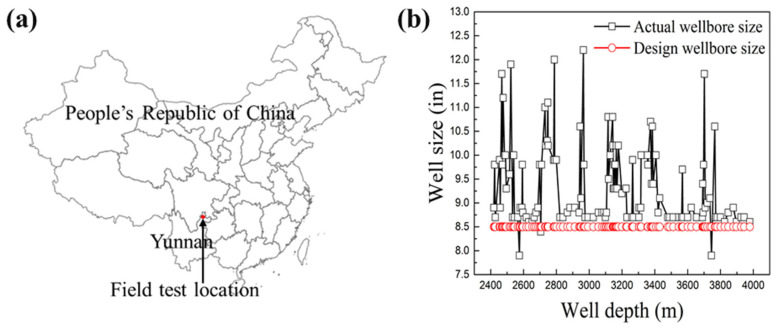
(**a**) Schematic map of the field test location. (**b**) The curve of the diameter of a drilled well in Yunnan, China.

Modified asphalt, 3% (*w*/*w*), was added to the drilling fluid and successfully applied to drill shale gas in Yunnan (Figure 12a) in a well depth of 2200–3980 m. The density of the drilling fluid was 2.0 g/cm^3^, and the temperature in the bottom hole was 102 °C. The stratigraphic lithology was as follows: grey-black mudstone, grey-black sandy limestone-bearing limestone, grey-black shale, argillaceous siltstone, and black carbonaceous shale.

As shown in Figure 12b, the average diameter expansion rate was 5.8%, and the diameter of the well was normal. No accidents occurred during the drilling of the well, the electrical test was successful, the operations of lifting and lowering the casing were smooth, and the cementing quality in the horizontal section reached 100%.

## 4. Conclusions

In this study, we have explored the potential application of modified asphalt as a shale inhibitor for water-based drilling fluid. The inhibition performance test showed that conventional inhibitors such as sodium chloride, potassium chloride, and potassium formate cannot effectively inhibit the hydration expansion of the shale formation with illite as the main clay mineral in Yunnan. After the shale was impressed in a modified asphalt solution, the hydration expansion of Yunnan shale was significantly reduced, the shale recovery was effectively improved, the amount of water absorption was reduced, and the compressive strength of the shale was effectively maintained. Modified asphalt formed a hydrophobic membrane through a large amount of adsorption on the surface of shale, inhibiting shale hydration. At the same time, modified asphalt can reduce the entry of water into the shale through blocking the pores, micro-cracks, and cracks in the formation, and thus, further inhibit shale hydration expansion. After being added to the drilling fluid, modified asphalt can effectively reduce the API fluid loss and also the high-temperature and high-pressure fluid loss, which are reduced from 0.6 mL and 5.4 mL to 0.2 mL and 4.0 mL, respectively. The field test showed that modified asphalt had practical application value. This present study provided inspiration for the development of novel asphalt inhibitors.

## Figures and Tables

**Table 1:** Experimental equipment.

Equipment	Model	Graphic
Pulse attenuation permeameter	ULP-713, Beijing Yineng, Beijing, China	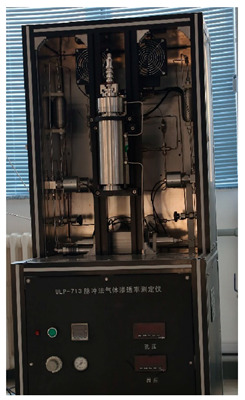
Core flow meter	LDY 50-180A, Jiangsu Hongbo, Nantong, China	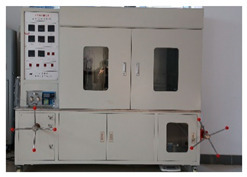
High-temperature and high-pressure dilatometer	HTP-C4, Qingdao Tongchun Petroleum Instrument, Qingdao, China	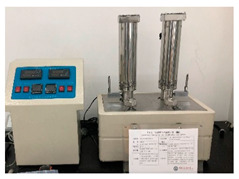
High-temperature roller heating furnace	GW300-PLC, Qingdao Tongchun Petroleum Instrument, Qingdao, China	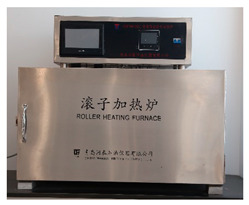
Molecular fluorescence spectrophotometer	RF-6000, Shimadzu Corporation, Kyoto, Japan	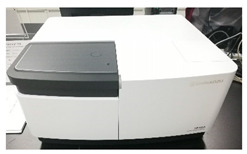
Optical contact angle meter	OCA-25, Dataphysics, Filderstadt, Germany	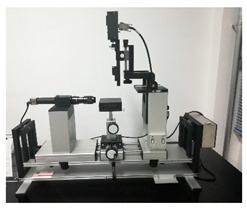
Rock triaxial tester	TAW-2000, Chaoyang Testing Instrument, Changchun, China	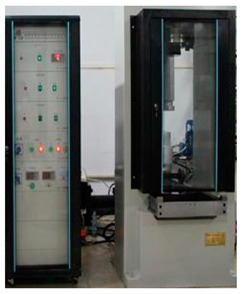

**Table 2:** Permeability of the shale before and after treatment with modified asphalt solution.

Sample	Initial Permeability K_0_ (10^−7^ μm^2^)	Concentration of Modified Asphalt	Permeability after Blocking K_f_ (10^−7^ μm^2^)	Blocking
1	20.46	water	38.52	/
2	53.63	0.5 wt %	20.13	62.47
3	41.37	1 wt %	9.97	75.90
4	25.78	2 wt %	5.35	79.25
5	31.25	3 wt %	2.63	91.58
6	18.67	4 wt %	0.58	96.89

**Table 3:** Drilling fluids composition.

Additive	Formula 1	Formula 2
Water	1000 kg	1000 kg
Bentonite	20 kg	20 kg
Na_2_CO_3_	2 kg	2 kg
Polyanionic cellulose	5 kg	5 kg
Polyethylene fluid loss additive	10 kg	10 kg
Modified asphalt	0	30 kg
Sulfonated brown coal resin	30 kg	30 kg
Lubricant	20 kg	20 kg

**Table 4:** Performance of drilling fluids.

Formula	Hot Rolling	Density (g/cm^3^)	PV (mPa·s)	YP (Pa)	Gel(G’/G”) (Pa/Pa)	FL_API_ (mL)	FL_HTHP_ (mL)
1	before	2.0	42	19	3.5/4	0.8	/
	after	2.0	41	20	3/3.5	0.6	5.4
2	before	2.0	43	20	4/4.5	0.4	/
	after	2.0	41	21	4/4.5	0.2	4.0

## Data Availability

Data available on request due to privacy.

## References

[1] Radwan A.E., Yin S., Hakimi M.H., Li H. (2023). Petroleum geology of conventional and unconventional resources: Introduction. Geol. J..

[2] Muther T., Qureshi H.A., Syed F.I., Aziz H., Siyal A., Dahaghi A.K., Negahban S. (2022). Unconventional hydrocarbon resources: Geological statistics, petrophysical characterization, and field development strategies. J. Pet. Explor. Prod. Technol..

[3] Hackley P.C., Warwick P.D. (2015). Unconventional Energy Resources: 2015 Review. Nat. Resour. Res..

[4] Kok M.V., Merey S. (2014). Shale Gas: Current Perspectives and Future Prospects in Turkey and the World. Energy Sources Part A-Recovery Util. Environ. Eff..

[5] Solarin S.A., Gil-Alana L.A., Lafuente C. (2020). An investigation of long range reliance on shale oil and shale gas production in the US market. Energy.

[6] Cotton M., Rattle I., Van Alstine J. (2014). Shale gas policy in the United Kingdom: An argumentative discourse analysis. Energy Policy.

[7] Wang J.S., Ryan D., Anthony E.J. (2011). Reducing the greenhouse gas footprint of shale gas. Energy Policy.

[8] Boyou N.V., Ismail I., Sulaiman W.R.W., Haddad A.S., Husein N., Hui H.T., Nadaraja K. (2019). Experimental investigation of hole cleaning in directional drilling by using nano-enhanced water-based drilling fluids. J. Pet. Sci. Eng..

[9] Akpan E.U., Enyi G.C., Nasr G., Yahaya A.A., Ahmadu A.A., Saidu B. (2019). Water-based drilling fluids for high-temperature applications and water-sensitive and dispersible shale formations. J. Pet. Sci. Eng..

[10] Skadsem H.J., Leulseged A., Cayeux E. (2019). Measurement of Drilling Fluid Rheology and Modeling of Thixotropic Behavior. Appl. Rheol..

[11] Chang Y., Huang R., Ries R.J., Masanet E. (2014). Shale-to-well energy use and air pollutant emissions of shale gas production in China. Appl. Energy.

[12] Wang C.-q., Lin X.-y., Mei X.-d., Luo X.-g. (2019). Performance of non-fired bricks containing oil-based drilling cuttings pyrolysis residues of shale gas. J. Clean. Prod..

[13] Villada Y., Iglesias M.C., Casis N., Erdmann E., Peresin M.S., Estenoz D. (2018). Cellulose nanofibrils as a replacement for xanthan gum (XGD) in water based muds (WBMs) to be used in shale formations. Cellulose.

[14] Xiang J., Zhu Y.M., Wang Y., Chen S.B., Jiang Z.F. (2022). Effect of Faults on Shale Pore Fracture and Shale Gas Preservation: A Case Study of the Wufeng-Longmaxi Formation in the Northeast Yunnan Area. Energy Fuels.

[15] Parizad A., Shahbazi K., Tanha A.A. (2018). Enhancement of polymeric water-based drilling fluid properties using nanoparticles. J. Pet. Sci. Eng..

[16] Van Oort E., Pasturel C., Bryla J., Ditlevsen F. (2019). Improved Wellbore Stability in North Sea Lark and Horda Shales Through Shale/Fluid-Compatibility Optimization. Spe Drill. Complet..

[17] Santiago V.-E., Jorge d.A.R.J., Ferreira d.A.C., Sandra Veiga N.R. (2021). Mechanism of shales stabilization by hydrophobized poly(ethylene glycol)/K^+^ in water-base drilling fluids. Pet. Explor. Dev..

[18] Elkatatny S., Jafarov T., Al-Majed A., Mahmoud M. (2019). Formation Damage Avoidance by Reducing Invasion with Sodium Silicate-Modified Water-Based Drilling Fluid. Energies.

[19] Murtaza M., Kamal M.S., Mahmoud M. (2020). Application of a Novel and Sustainable Silicate Solution as an Alternative to Sodium Silicate for Clay Swelling Inhibition. ACS Omega.

[20] Naeimavi M., Khazali F., Abdideh M., Saadati Z. (2021). Potassium sorbate left-to-right markas substitute for KCl to shale left-to-right markinhibition in water-base drilling fluids. Energy Sources Part A-Recovery Util. Environ. Eff..

[21] Zheng Y., Zaoui A. (2011). How water and counterions diffuse into the hydrated montmorillonite. Solid State Ion..

[22] Fleming N., Moland L.G., Svanes G., Watson R., Green J., Patey I., Byrne M., Howard S. (2016). Formate Drilling and Completion Fluids: Evaluation of Potential Well-Productivity Impact, Valemon. SPE Prod. Oper..

[23] Wang X., Yin J., Xu J., Bu W., Sun L., Wang J., Jing Y., Ren Y., Sun Y. (2023). Development and Function Mechanism of Intercalation Adsorption Inhibitor with High Temperature Resistance. Oilfield Chem..

[24] Oseh J.O., Norddin M.N.A.M., Muhamad H.N., Ismail I., Gbadamosi A.O., Agi A., Ismail A.R., Blkoor S.O. (2020). Influence of (3-Aminopropyl) triethoxysilane on entrapped polypropylene at nanosilica composite for shale swelling and hydration inhibition. J. Pet. Sci. Eng..

[25] Xu J.-g., Su K., Li M., Lyu X., Zhu S., Huang Y. (2023). Hydration inhibition and physical plugging to enhance shale stability using zwitterionic polymer/nano-silica composite. J. Mol. Liq..

[26] Huber J., Plank J., Heidlas J., Keilhofer G., Lange P. (2009). Additive for Drilling Fluids. United. States Patent.

[27] Xiong Z.Q., Tao S.X., Li X.D., Shan W.J., Dong H.Y. Development and Application of Anti-Collapse & Anti-Drag Agent for Drilling Fluid. Proceedings of the International (China) Geological Engineering Drilling Technology Conference (IGEDTC).

[28] Xionghu Z., Egwu S.B., Jingen D., Liujie M., Xiangru J. (2022). Synthesis of Asphalt Nanoparticles and Their Effects on Drilling Fluid Properties and Shale Dispersion. Spe Drill. Complet..

[29] Rasool M.H., Ahmad M., Ayoub M., Abbas M.A. (2023). A Novel Ascorbic Acid Based Natural Deep Eutectic Solvent as a Drilling Mud Additive for Shale Stabilization. Processes.

[30] Jin J.F., Sun J.S., Lv K.H., Hou Q.L., Guo X., Liu K.S., Deng Y., Song L.D. (2023). Catalytic pyrolysis of oil shale using tailored Cu@zeolite catalyst and molecular dynamic simulation. Energy.

[31] Jin J.F., Sun J.S., Lv K.H., Guo X., Hou Q.L., Liu J.P., Wang J.T., Bai Y.R., Huang X.B. (2022). Oxygen vacancy BiO_2_-x/Bi_2_WO_6_ synchronous coupling with Bi metal for phenol removal via visible and near-infrared light irradiation. J. Colloid Interface Sci..

[32] Cao J., Meng L., Yang Y., Zhu Y., Wang X., Yao C., Sun M., Zhong H. (2017). Novel Acrylamide/2-Acrylamide-2-methylpropanesulfonic Acid/4-Vinylpyridine Terpolymer as an Anti-calcium Contamination Fluid-Loss Additive for Water-Based Drilling Fluids. Energy Fuels.

[33] Ramos-Tejada M.M., Galindo-Gonzalez C., Perea R., Duran J.D.G. (2006). Effect of charged polyelectrolytes on the electrophoretic behavior, stability, and viscoelastic properties of montmorillonite suspensions. J. Rheol..

